# The effect of Ulinastatin on postoperative course in cardiopulmonary bypass patients in Asia: a meta-analysis of randomized controlled trials

**DOI:** 10.1186/s13019-022-01811-z

**Published:** 2022-04-05

**Authors:** Hu Zhenyu, Yuan Qiaoli, Chen Guangxiang, Wang Maohua

**Affiliations:** 1 Department of Anesthesiology, Laboratory of Anesthesiology, The Affiliated Hospital of Southwest Medical University, Luzhou, People’s Republic of China; 2 Department of Radiology, The Affiliated Hospital of Southwest Medical University, Luzhou, People’s Republic of China

**Keywords:** Ulinastatin, Cardiopulmonary bypass, Clinical outcome, ICU length of stay, Prognosis, Acute inflammatory disorder

## Abstract

**Objectives:**

To evaluate the effect of urinary trypsin inhibitor (UTI) or Ulinastatin on postoperative course and clinical outcomes in patients with cardiopulmonary bypass.

**Methods:**

We searched PubMed, Embase, Web of Science, and Cochrane Library for the keywords UTI and Cardiopulmonary bypass (CPB). The primary outcome measure was the intensive care unit length of stay (ICU LOS), and results were stratified for relevant subgroups (dosage of UTI). The effects of UTI on mechanical ventilation duration (MVD), hospital LOS, renal failure incidence (RFI), and all-cause mortality were studied as secondary outcomes.

**Results:**

Twelve randomized controlled trials (enrolling 1620 patients) were evaluated. Eleven studies pooled for subgroup analysis showed that using UTI persistently or with a considerable amount would lead to a shorter ICU LOS (95% CI, − 0.69 to − 0.06; *P* = 0.0001). Ten studies showed that UTI could shorten MVD in patients (95% CI, − 1.505 to − 0.473; *P* < 0.0001). RFI generally showed a more favourable outcome with UTI treatment (95%CI, 0.18–1.17; *P* = 0.10). And the current evidence was insufficient to prove that UTI could reduce the hospital LOS (95% CI, − 0.22 to 0.16; *P* = 0.75) and the all-cause mortality rate (95% CI, 0.24–2.30; *P* = 0.60).

**Conclusions:**

Various subsets of UTI treatment suggested that UTI could shorten ICU LOS, and it is associated with the dosage of UTI. Considering the substantial heterogeneity and lack of criteria for UTI dosage, more evidence is needed to establish a standard dosing guideline.

## Introduction

Cardiopulmonary bypass (CPB) has been used for most cardiac surgeries for over half a century worldwide. CPB has been shown to provoke ischemia–reperfusion and subsequent cellular injury with inflammatory reaction [1]. Moreover, shear stress associated with cardiopulmonary bypass could induce the expression of inflammatory cytokines and necroptosis in monocytes [2].

On account of the CPB-induced damage, up to 10% of patients require prolonged postoperative care [3], with a more extended intensive care unit length of stay (ICU LOS) and mechanical ventilation duration (MVD) [4]. Recently, ICU-acquired weakness is evolving into a complication that cannot be neglected [5], and It is estimated that the ICU consumes 20%of hospital expenditures and approximately 1%of gross domestic product[6, 7]. Furthermore, patients with a long MVD often suffer many complications, such as ventilator-associated pneumonia [8] and pressure sores [9]. All of these resulted in the use of an incredible amount of medical resources. Hence, patients who underwent CPB are associated with a higher healthcare cost [10, 11].

Urinary trypsin inhibitor(UTI) or Ulinastatin was first identified in human blood, urine and other tissues in the 1980s [12], with inhibitory effects on a variety of proteases [13]. UTI has been testified to have the potential of reducing CPB-induced damage[14] and in turn may shorten ICU LOS and MVD. Based on recent studies, UTI not only could inhibit the activation of various pro-inflammatory cytokines but may also inhibit the release of neutrophil elastase [15]. Meanwhile, other studies have found that UTI attenuates inflammation and resists oxidative stress by inhibiting the JNK/NF-kappaB signalling pathway and PI3K/Akt/Nrf2 pathway [16, 17]. Besides, UTI could afford a certain degree of protection in alleviating cerebral ischemia–reperfusion injury by activating the Nrf-2/HO-1 signalling pathway and may improve myocardial ischemia–reperfusion injury through endoplasmic reticulum stress-induced apoptosis pathway [18, 19]. Furthermore, as a natural anti-inflammatory molecule, UTI could have excellent prospects in both pharmacologic prophylaxis of post-endoscopic retrograde cholangiopancreatography pancreatitis and the treatment of severe decompression sickness, and even in the therapy of SARS-CoV-2 infection [20–22]. In the regions of Asia, UTI is used as an essential drug to treat acute inflammatory disorders, including pancreatitis, sepsis and shock [23, 24].

Recent clinical trials conducted on CPB patients with UTI showed a beneficial trend in restraining inflammation[25]. And five meta-analyses on this topic have been published [26–30]; among which, one [26] evaluated the effect of UTI in reducing postoperative bleeding of CPB patients and did not analyze other clinical outcomes; four [27–30] analyzed the effect of UTI, but no significant benefit in reducing ICU LOS and MVD had been shown. More to the point, a retrospective study indicated that UTI did not improve postoperative outcomes in CPB patients [31]. It’s difficult to interpret why UTI is beneficial for reducing inflammation, but could not improve other clinical outcomes. Therefore, this meta-analysis was conducted to clarify the clinical effectiveness of UTI in CPB patients.

## Materials and methods

According to the PRISMA (Preferred Reporting Items for Systematic Reviews and Meta-Analysis) statement for reporting systematic reviews and meta-analyses [29], we conducted the meta-analysis with the registration number CRD42020215640 (registered on 21 November 2020). Two groups of researchers (Hu Zhenyu and Yuan Qiaoli) independently conducted literature searches, established the study inclusion and exclusion criteria, performed quality assessment, and extracted data. Any disagreement between the two researchers was resolved by the senior authors (Wang Maohua). Eligibility criteria included randomized controlled trials (RCTs) which assessed the effect of UTI treatment on clinical outcomes in CPB patients.

### Data sources and searches

The electronic databases PubMed, EMBASE, Web of Science, and Cochrane library were searched systematically (20 January 2022) with the following search strategy:”ulinastatin” OR “UTI68″ OR “acid-stable protease inhibitor” OR “MR 20 (magnetic powder) of urinastatin” AND "Cardiopulmonary Bypass" OR “cardiopulmonary”. Besides, personal records and reference lists of relevant articles were screened. We tried to contact the corresponding authors if information about important clinical outcome indicators was missing, but sadly there was no response.

### Study selection

Studies were independently screened based on title and abstract by two authors (Hu Zhenyu and Yuan Qiaoli), and differences were resolved by consensus. Study inclusion criteria were: (1) patients who underwent surgery with CBP; (2) type of interventions: used UTI alone regardless of treatment duration; (3) research design: RCTs only. Exclusion criteria included: (1) pediatric studies; (2) review articles, case reports and letters; (3) animal experiment studies; (4) duplicate publications; (5) studies that did not describe correlation outcomes of interest. The eligibility of the remaining studies for final inclusion was further determined by reading the full text.

### Data synthesis and analysis

Data from individual clinical studies adopting an intention to treat design were used for this meta-analysis. A random-effects model that used the DerSimonian and Laird method was applied for all individual pooling estimates. For dichotomous outcomes (e.g., renal failure), the selected effect size was risk ratio (RR) with 95% confidence interval (CI) calculated from the two-by-two table. For continuous outcome (i.e., ICU LOS), the effect size was standard deviation (STD) because the constant outcome units were miscellaneous in the included studies. The median (quartile interval) data were conversed to mean ± standard deviation using Shi et.al.’s methods [32], so all continuous data were expressed as mean ± standard deviation.

Heterogeneity was assessed by using the I^2^ statistics, chi-square test, Tau^2^, and visualization in a funnel plot. The effects of UTI were independent of many confounding factors. To explore heterogeneity, the subgroup design was based on the dosage of UTI and analyzed using a meta-regression framework. We performed sensitivity analyses to explore the potential impact of a single trial on the overall results by omitting one trial from the meta-analysis at a time. Studies were conducted with Review Manager software 5.4 and Statistic/Data Analysis 12.0.

## Results

### Search results and study characteristics

**Fig. 1:**
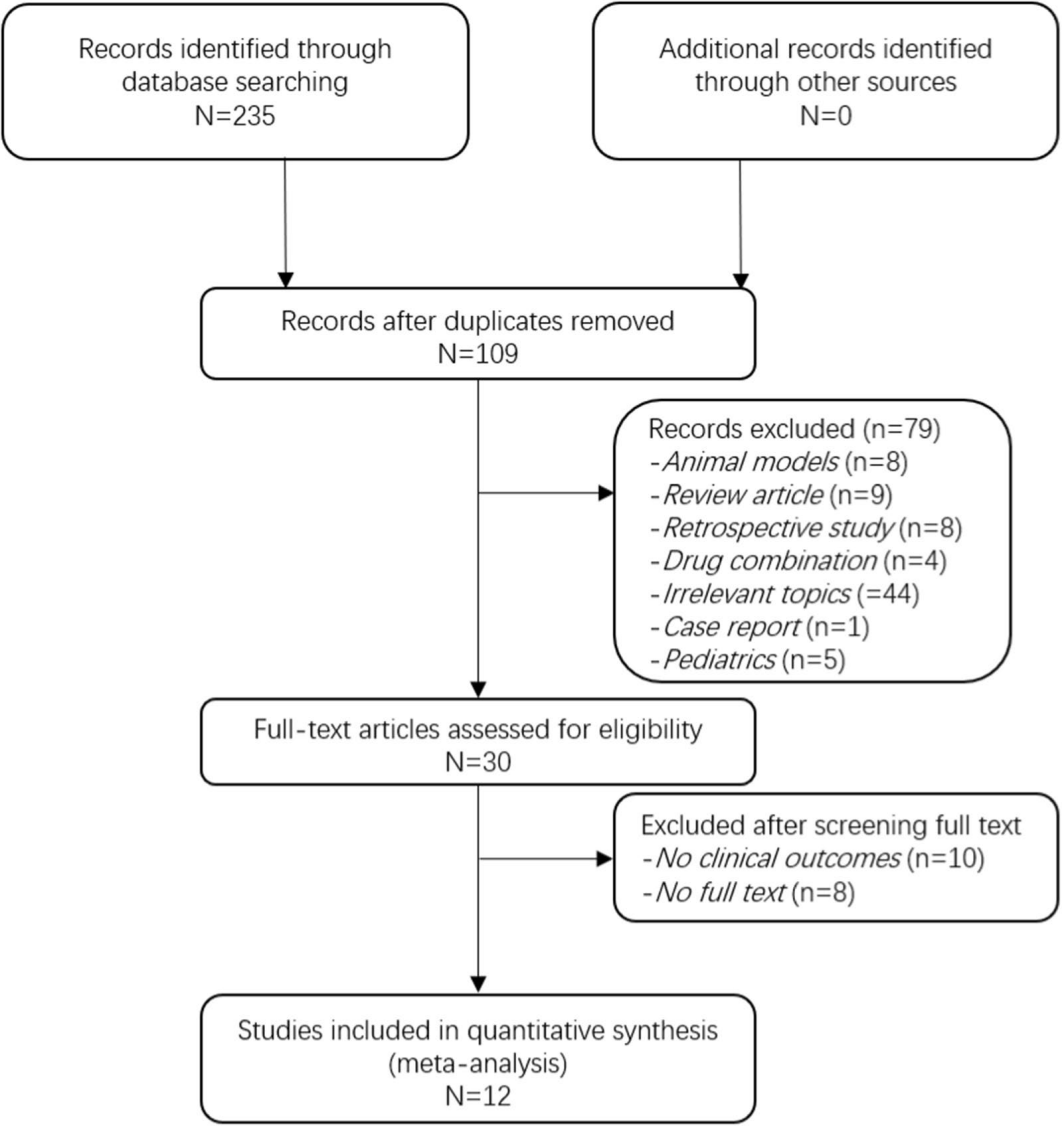
Meta-analysis flowchart for selecting eligible studies

Figure 1 is the PRISMA flow diagram that summarizes the search and selection strategy. Our search strategy resulted in considering 235 studies for inclusion. After removing duplicates, 109 reviews were screened for titles and abstracts, and 58 studies were excluded. Finally, 51 full-text articles were assessed for eligibility. In total, twelve RCTs were included for this meta-analysis, of which one study [33] was fit only for secondary outcomes. Due to the data of ICU LOS were described as median (quartile interval) in this article, it’s hard to combine them with other data. We had tried to contact the authors for complete data, yet no response was heard. The included articles were published from 2006 to 2020 [33–44], and data collection was performed between 1998 and 2016. Three studies included patients who underwent coronary artery bypass graft (CABG) surgery, four included patients with valve replacement or valve repairment, three included patients receiving combined surgery (valve relevant surgery and CABG) which was collectively called open-heart surgery, and the remaining two studies included aortic arch replacement (AAR) patients. No adverse event existed among these studies. The population characteristics of included patients were summed up in Table1.

**Table 1:** Characteristics of the clinical trials included in the meta-analysis

Reference, years, country of origin	Number of patients		Mean age		Gender (F/M)		Surgery	Interventions		CPB time (min)		Adverse effects	
	UTI group	Control group	UTI group	Control group	UTI group	Control group		UTI group	Control group	UTI group	Control group		
Zhang et al. 2020 China	142	141	49.0 ± 14.3	50.3 ± 12.4	63/79	71/70	Open heart surgery	1,000,000 U iv, the half after anesthesia induction and the rest prime into CPB	Equivalent normal saline	102.87 ± 42.7	98.69 ± 47.76	No	
Xu et al. 2017 China	20	20	53.4 ± 5.2	53.2 ± 5.1	12/8	13/7	Open heart surgery	5000 U/kg iv before CPB and at 1–3 days postoperatively	Equivalent normal saline	Unclear		No	
Wang et al. 2016 China	36	30	48.4 ± 11.3	49.3 ± 11.7	32/4	26/4	AAR	300,000 U/8 h iv before 3 days postoperatively and 300,000 U/2 h during surgery	NR	228.6 ± 69.1	22.4 ± 62.5	No	
Pang et al. 2015 China	30	30	53.5 ± 9.8	50.2 ± 9.1	15/15	21/9	Open heart surgery	5000 U/Kg iv after anesthesia induction	Equivalent normal Saline	215.3 ± 57.9	221.6 ± 76.1	No	
Xu et al. 2013 China	18	18	54.8 ± 8.9	53.2 ± 7.3	16/2	15/3	AAR	20,000 U/Kg iv after anesthesia induction	Equivalent placebo	235.5 ± 25.9	247.2 ± 20.3	No	
Hao et al. 2013 China	20	20	56.36 ± 10.81	54.27 ± 10.90	22/18		MVR, DVR, AVR	1000 U/Kg iv during the preoperative period	NR	98.36 ± 9.87	105.55 ± 44.16	No	
Chen et al. 2012 China	30	30	49.8 ± 10.8	50.4 ± 10.0	14/16	12/18	VR	12,000 U/Kg iv after anesthesia induction	Equivalent normal Saline	102.5 ± 20.7	105.9 ± 20.2	No	
Oh et al. 2012 Korea	30	30	67 ± 19	60 ± 12	20/10	16/14	AVR	1,000,000 U iv during surgery	Equivalent normal Saline	99 ± 25	96 ± 22	No	
ONG et al. 2011 Korea	24	24	51.9 ± 17.3	52.7 ± 18.9	8/16	9/15	Aortic valve repair	5000 U/Kg iv before CPB	Equivalent normal Saline	164.3 ± 31.5	173.4 ± 28.4	No	
Zhou et al. 2010 China	20	20	61.1 ± 8.7		28/12		CABG	15,000 U/Kg iv during surgery	Equivalent normal Saline	83.5 ± 23.1	79.6 ± 25.7	No	
Jiang et al. 2007 China	15	15	57.4 ± 6.6	56.8 ± 6.1	11/9	11/9	CABG	1,000,000 U iv during surgery	Equivalent placebo	108 ± 29	117 ± 34	No	
Nakanishi et al. 2006 Japan	14	14	62 ± 9	61 ± 10	12/2	11/3	CABG	5000 U/Kg iv before aortic cannulation for CBP	Equivalent normal Saline	150 ± 37	135 ± 39	No	

CBP = Cardiopulmonary Bypass; TAR = Total aortic arch replacement; AAR = Aortic Arch Replacement; VR = Valve Replacement, including Aortic Valve Replacement, Mitral Valve Replacement; MVR = Mitral Valve Replacement; DVR = Double (aortic and mitral) Valve Replacement; AVR = Aortic Valve Replacement; CABG = Coronary Artery Bypass Graft

### Data extraction and quality assessment

**Fig. 2:**
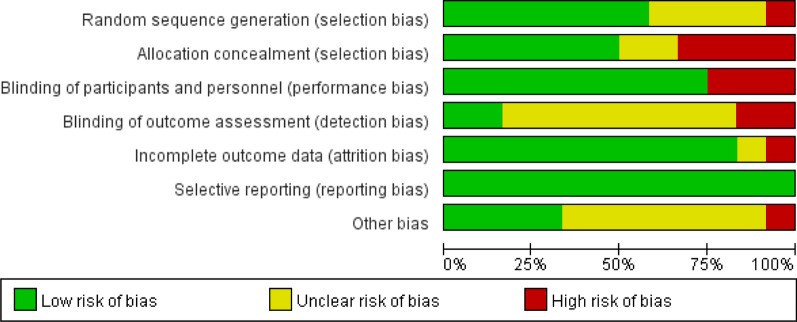
Risk of bias of included studies

**Fig. 3:**
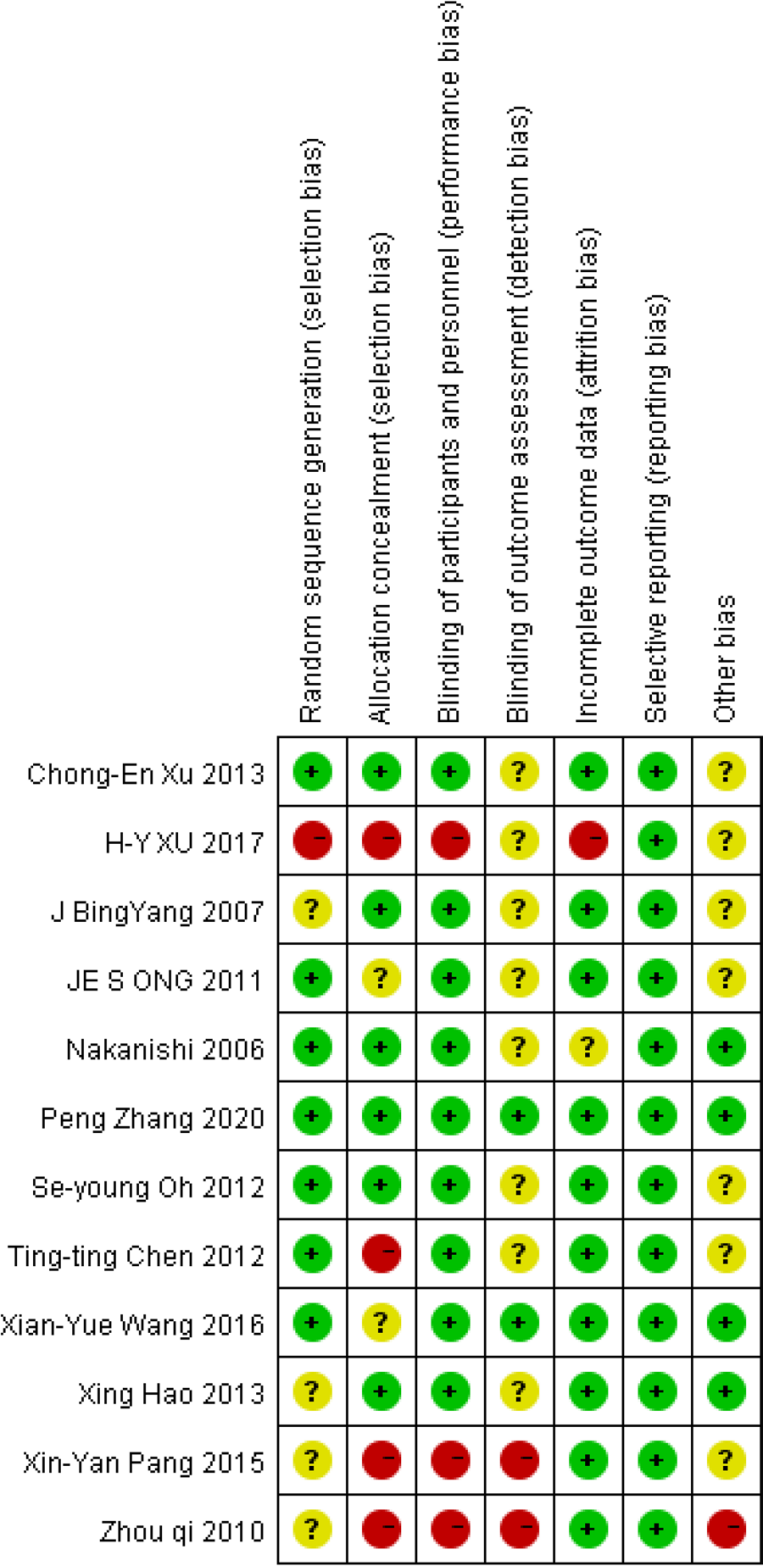
Risk of bias summary

Relevant data were extracted using a standardized data extraction sheet. The primary outcome measure was ICU LOS. MVD, hospital LOS, and renal failure incidence (RFI) were also noted as secondary outcomes. The research methodology quality of each RCT was assessed following the Cochrane Risk of Bias (RoB 2.0) Tool for randomized controlled trials [45]. The Cochrane tool assesses five domains, including bias arising from the randomization process, deviations from intended interventions, missing outcome data, measurement of the outcome, and selecting the reported result. For each domain, bias was classified as either low, uncertain, or high. The risk of bias of included studies and the overall risk of bias are shown in Figs. 2 and 3, respectively. Furthermore, the studies are substantially different in methods in terms of study population and study design. Hence, results were stratified and, if possible, analyzed for subsets. Two authors independently screened, reviewed, and scored each trial using this method and extracted data for analysis. Disagreements about scoring or extracting data were resolved through discussion or consulting with a third expert.

### Outcomes

#### ICU length of stay

**Fig. 4:**
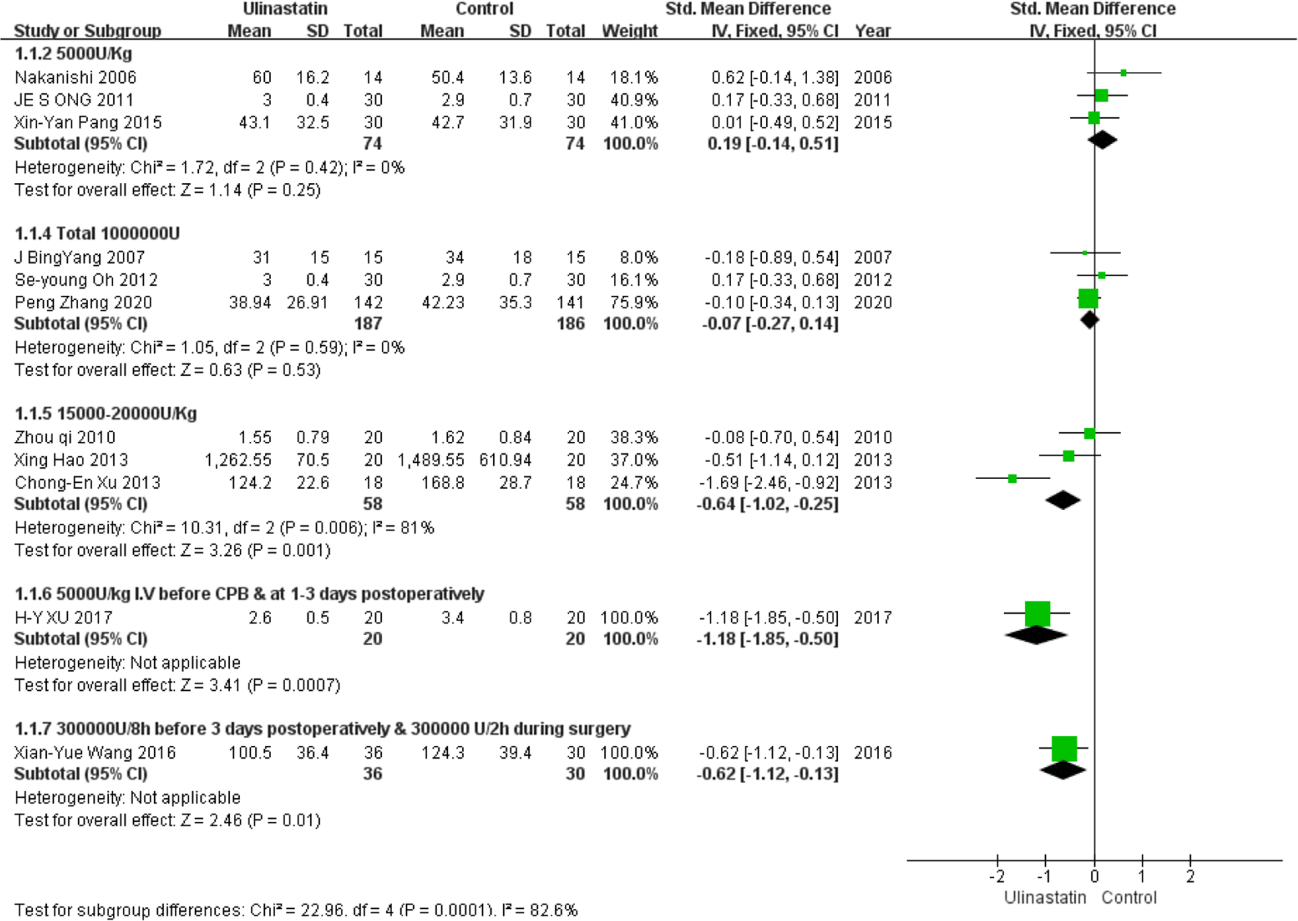
Forest plots of ICU LOS in the control groups and UTI groups. CI = confidence interval; IV = inverse variance; Std = standardized mean difference

**Fig. 5:**
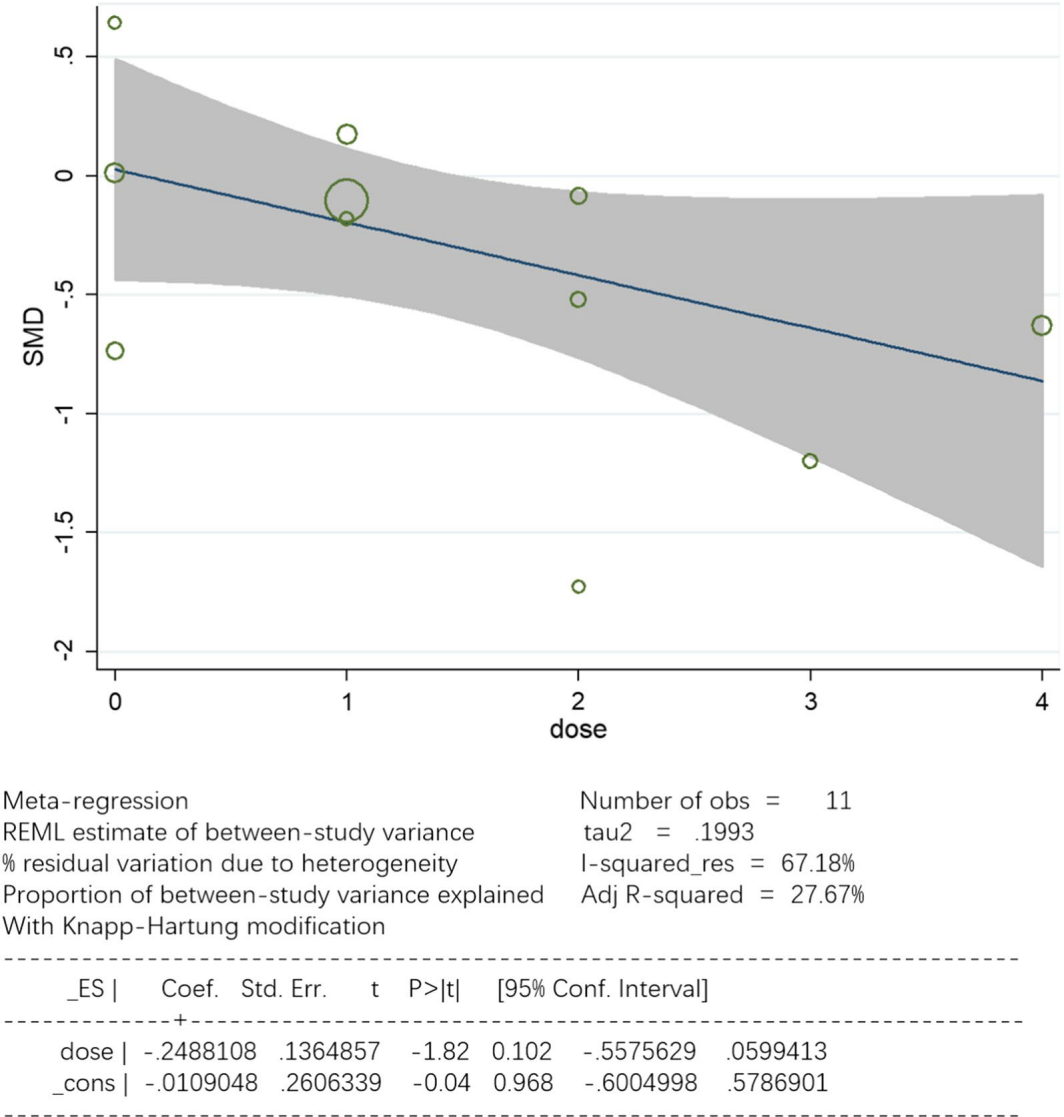
Meta-regression analysis of effects on ICU LOS by dosages of UTI

Eleven studies with a total of 743 patients, including 375 in the control group and 368 in the UTI group, were available for analysis. A forest plot is presented in Fig. 4. Pooled analysis showed that the control groups and UTI groups differed significantly in ICU LOS (95% CI, − 0.69 to − 0.06; *P* = 0.0001). The heterogeneity among studies was conspicuous(*I*^2^ = 82.7%). Based on different UTI dosages and methods of administration, a single factor regression analysis was used. As shown in Fig. 5, the result indicates that UTI dosage might be one of the sources of heterogeneity (tau2 = 0.1993; I-squared res = 67.18%; Adj R-squared = 27.67%; *P* = 0.102).

#### Mechanical ventilation duration

**Fig. 6:**
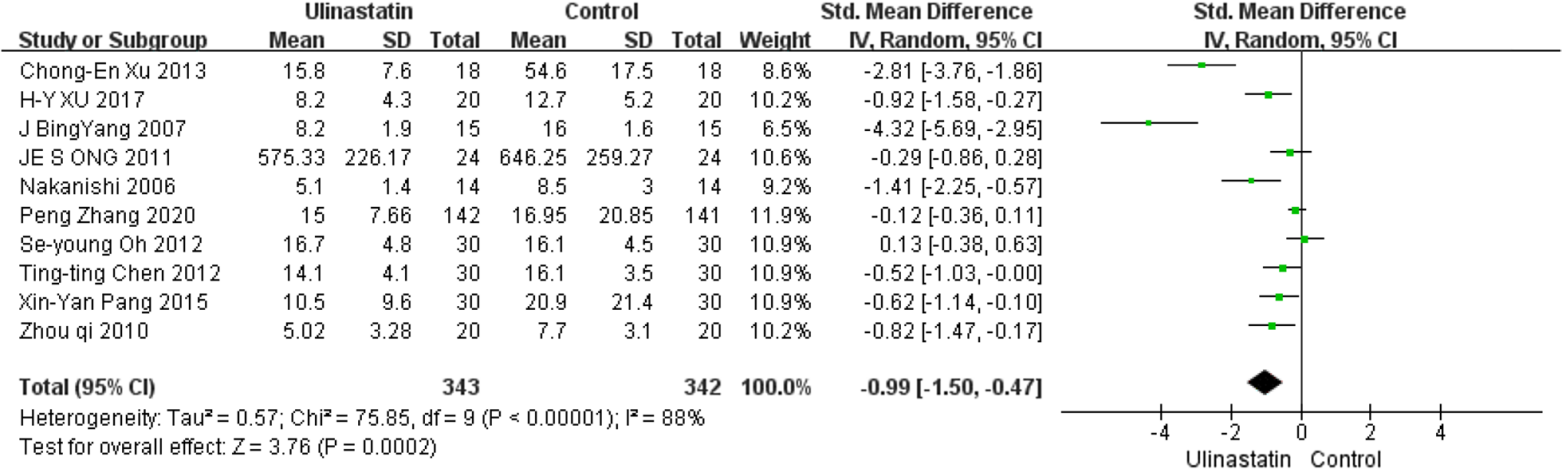
Forest plot for MVD in the control groups and UTI groups

Ten studies enrolling 685 patients, including 343 in the UTI group and 342 in the control group, analyzed MVD. A forest plot is presented in Fig. 6, which shows a significantly longer ventilation time in the Control group than in the UTI group (95% CI, − 1.505 to − 0.473; *P* < 0.0001). Pooled data synthesis of this outcome showed marked heterogeneity(I^2^ = 88.1%).

#### Hospital length of stay

**Fig. 7:**
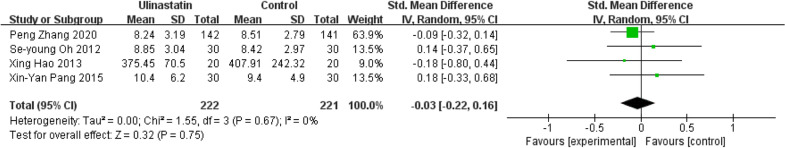
Forest plot for hospital LOS in the control groups and UTI groups

Four studies (443 patients) including 222 in the UTI group and 221 in the control group, analyzed hospital LOS. A forest plot is presented in Fig. 7, and it seems that the evidence was insufficient to prove the efficiency of UTI in shortening hospital LOS (95% CI, − 0.22 to 0.16; *P* = 0.75). Pooled data synthesis of this outcome showed no heterogeneity(I^2^ = 0%).

#### Renal failure incidence

**Fig. 8:**
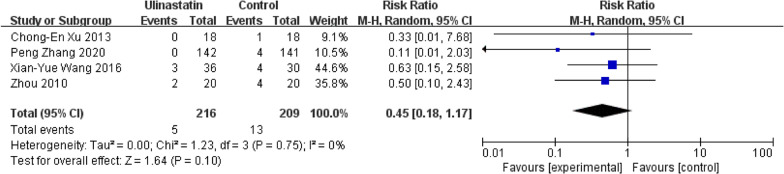
Forest plot for RFI in the control groups and UTI groups

Four studies enrolling 385 patients were taken in the analysis of RFI. Pooled analysis showed a significantly higher rate of renal failure in the control group than in the UTI group (95% CI, 0.24–2.30; *P* = 0.10) with no heterogeneity(I^2^ = 0%). The results are shown in Fig. 8.

#### All-cause mortality rate

**Fig. 9:**
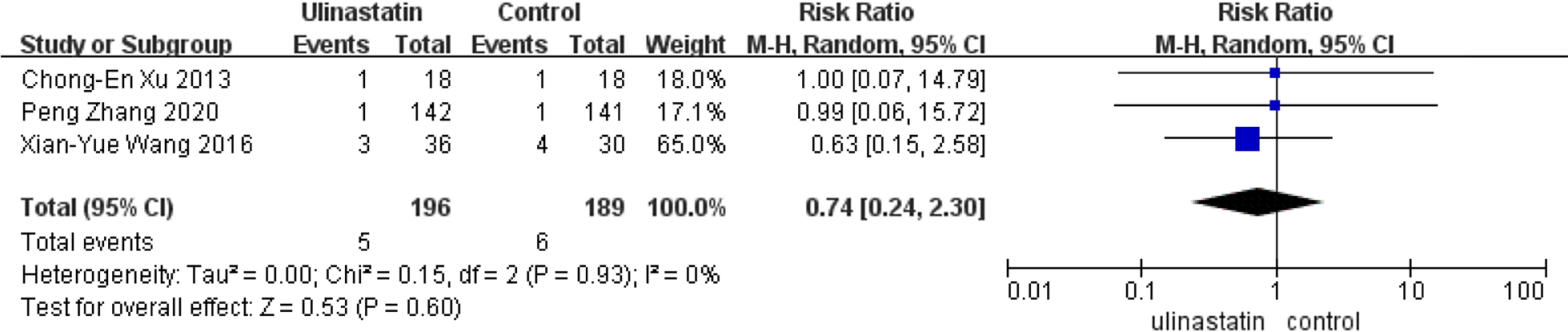
Forest plot for all-cause mortality rate in the control groups and UTI groups

Only three studies reported the all-cause mortality rate [34, 36, 38]. So We took the three studies enrolling 385 patients into the analysis of all-cause mortality. As presented in Fig. 9, the current evidence could not prove the efficiency of UTI in reducing the all-cause mortality rate (95% CI, 0.24–2.30; *P* = 0.60). Pooled data synthesis of this outcome showed no heterogeneity (I^2^ = 0%).

#### Publication bias and sensitivity analysis

**Fig. 10:**
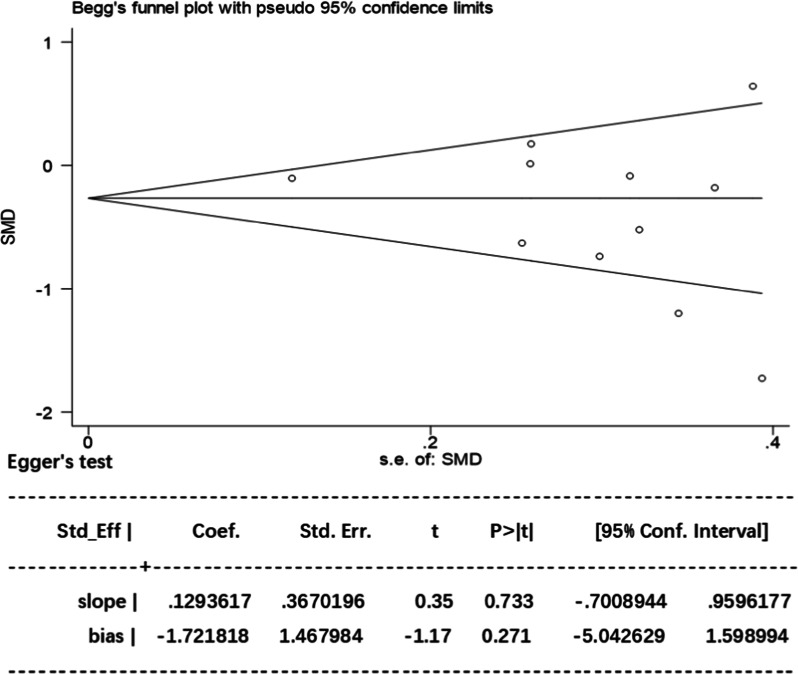
The Begg’s test and Egger’s test for ICU LOS. Begg’s test: rank correlation test; Egger’s test: linear regression method; SMD: standardized mean difference; 95% CI confidence interval

Unmeasured bias may impose a further limitation inherent to analyses in these RCTs. All the included studies involved the use of UTI for treating CPB patients. Using ICU LOS as the primary variable, the included studies were evaluated for the effect of study size. The funnel plot demonstrated an approximate symmetrical shape, suggesting that substantial publication bias is remote. Besides, Egger’s test also revealed a statistically substantial symmetry (*p* = 0.733). Therefore, potential publication bias had no significant influence on the results (Fig. 10).

**Fig. 11:**
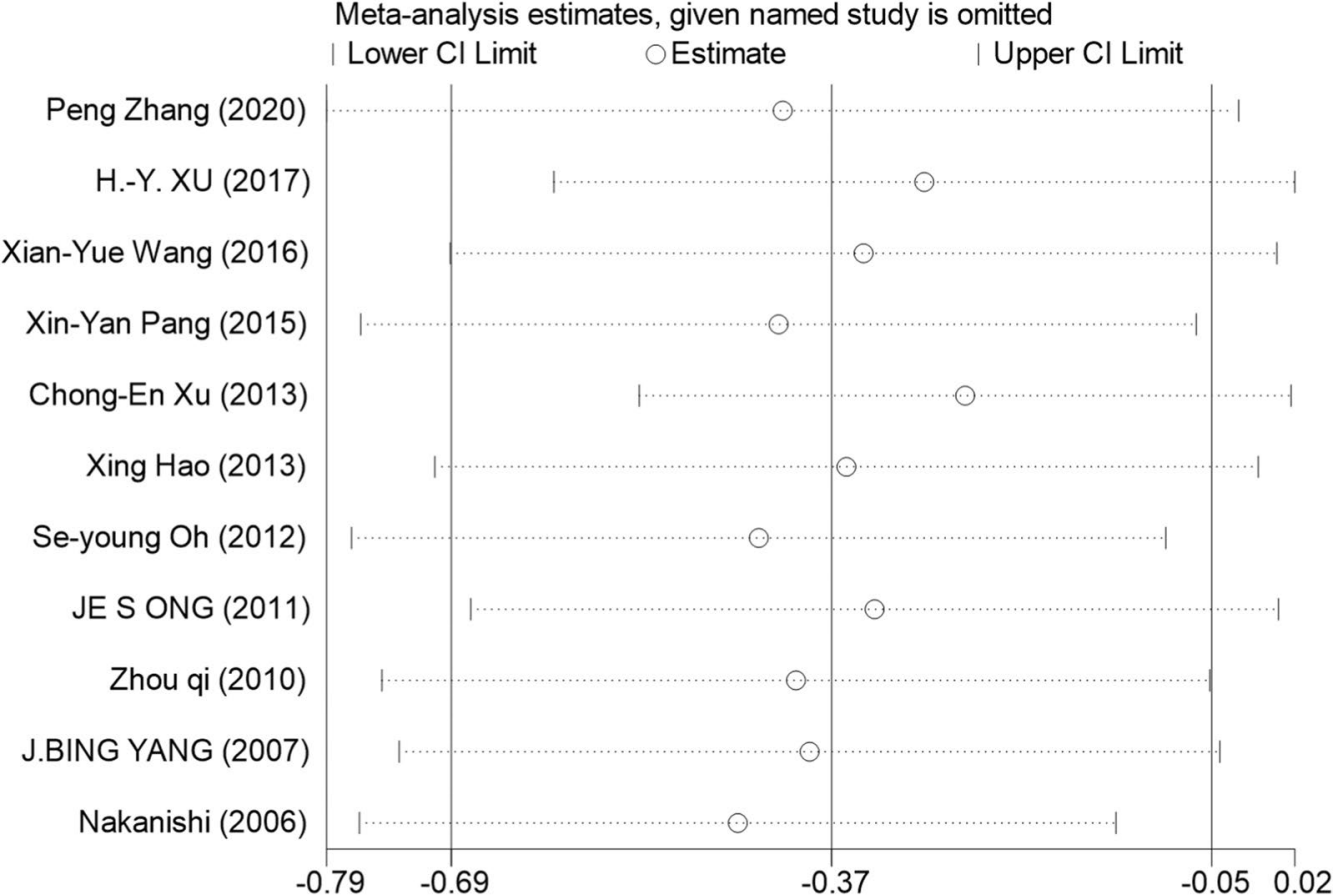
The form of sensitivity analysis

As to ICU LOS, sensitivity analysis showed that omitting specific trails had no significant impact on this outcome (Fig. 11). We could not entirely rule out the possibility that our findings are impacted by publication bias from the funnel plot. Three studies were outside of the confidence interval in Begg's funnel plot with pseudo 95% confidence limits. And we believe that the high heterogeneity may arise from many factors such as sample size, different design methods and study population.

## Discussion

Commonly associated with systematic inflammatory, cardiac surgery with CPB is inimical to clinical outcomes of the patients [46]. Meanwhile, Ulinastatin, a protease inhibitor emanating from human urine and blood, has potent anti-inflammatory and anticoagulant activity [47] which has been shown to ameliorate inflammatory response to CPB [22] significantly. And several immunomodulatory strategies for UTI have been evaluated accordingly. Nonetheless, what is much less clear is the impact of UTI on the postoperative course and overall outcomes in CPB patients [48].

This meta-analysis identified 11 RCTs investigating the effect of UTI on ICU LOS of CPB patients. Despite the limitations of RCTs included in this meta-analysis and the existence of considerable heterogeneity, with data pooled from all these patients, we could still see that UTI could be associated with ICU LOS. After meta-regression was analyzed, we discovered the source of heterogeneity might be UTI dosage, MVD was found to be associated with UTI too. However, considering the significant heterogeneity, profound conclusions and causal inferences had been hampered.

The methodology used in include studies may substantially influence our findings. What’s more, UTI dosage, age range, complications of patients and other confounders might also be the heterogeneity source. According to the meta-regression analysis, UTI dosage may be the most critical factor determining the effect size (I-squared_res = 67.18%). In our finding, three studies[38, 39, 42] used a higher dosage (15,000–20,000 U/Kg) and two studies (5000U/Kg I.V *before CPB & at 1-3 days postoperatively* [35] *and* 300,000U/8 h *from admission to* 3 days* postoperatively &* 300,000 U/2 h *during surgery* [36]) used a considerable amount of UTI persistently, and based on subgroup analysis, all of them effectively reduced ICU LOS. So we find that maintaining the blood concentration of UTI is critical for CPB patients, as giving a high dose at one time or giving amounts of dose over a period of time for CPB patients could achieve better clinical outcomes, but the best dosage warrants further investigation. Previous studies explored the effects of different dosages of UTI on CPB patients, and a study [49] compared the effect of 5000U/Kg UTI to 20,000U/Kg UTI on pulmonary protection after CPB, and results showed a better outcome with the higher dose of UTI. Another study [50] established three different UTI dosage groups (20,000 U/Kg, 40,000 U/Kg, 60,000 U/Kg) and one control group (100,000 U *during surgery &* 100,000U/8 h *postoperatively for* 2 days)*.* They discovered that the inflammatory cytokine levels were lower in the control group than in the 20,000 U/Kg group, and 60,000 IU/Kg of UTI exhibited the lowest inflammatory cytokine levels. These results were in line with our findings and could be associated with UTI pharmacokinetics. UTI is mainly eliminated in the kidneys with a half-life of about 33 min [51], while the duration of CPB often exceeds 40 min and activation of leukocytes and release of proinflammatory factors peak at 4-6 h after surgery[45]. Nevertheless, the study by Nakanishi et al. [44] contradicts most of our other findings. It is possibly an outlier, as it is the only study that came from Japan with a sample size that is too small (n = 28).

Our results regarding MVD should be interpreted with caution because of the high heterogeneity (*I*^2^ = 88.1%). Owing to the discrepancies in the definition of MVD, the high heterogeneity in ventilation time may be artificial, similar to ICU LOS, so we need more studies to analyse factors affecting the results. Therefore, the marked heterogeneity among the studies impeded the drawing of a powerful conclusion.

Our results about RFI shows that four studies with a marginal significance (*P* = 0.10, *I*^2^ = 0%) recorded RFI, which means that UTI treatment could protect renal function in CPB patients, this finding is consistent with a retrospective study [52]. previous studies [53, 54] proved that the systematic inflammatory response syndrome in CPB plays a vital role in the development of RFI, and other studies had found a correlation between UTI treatment and multiple organ function protection [55].

Our study could not prove that hospital LOS is related to UTI treatment. Four studies (443 patients) considered hospital LOS, but the sample was too small to have a high confidence level, although the heterogeneity was negligible (*I*^2^ = 0%). And our study on the all-cause mortality rate did not show a significant effect on UTI treatment.

### Limitation

Some limitations in this meta-analysis warrant further consideration. Firstly, an additional restriction inherent to analyses is that some short reviews concerning UTI treatment and ICU LOS with negative results may not be submitted or accepted for publication. Therefore, the number of studies included in this meta-analysis is quite small. Secondly, the definition of clinical outcomes was not consistent among these studies, which may substantially influence our findings:

1. ICU LOS: only one study [41] described their ICU discharged standard;
2. MVD: seven of the included studies defined this parameter as the postoperative duration of mechanical ventilation [34, 37, 38, 40–43], and the other three circumscribed it as extubation time with no declaration of whether it is postoperative or not [33, 35, 44];
3. RFI: two studies [36, 38] described their RFI as”Postoperative renal failure needing ultrafiltration”, one described RFI as “Renal dysfunction according to blood routine results” [42], and the last one did not clarify how RFI was defined [34];
4. The rest of the clinical outcomes have no definite criterion.

These differences in methodology may conduce to heterogeneity. Thirdly, there is a lack of sufficient evidence to prove that UTI treatment could shorten hospital LOS or reduce the all-cause mortality rate. In the absence of studies specifically addressing the effects of UTI in other surgeries, such as dissecting aneurysms of ascending aorta, heart or lung transplantation, the vast majority of current analyses consist of patients with CABG, valve replacement, AAR, and combined surgery. Fourthly, large RCTs with a patient population of more than 1,000 have traditionally been considered the “ gold standard” for evaluating the reliability of clinical interventions [56], ergo hindered by the small sample size in included studies, a large number of high-quality RCTs are called for. Lastly, since UTI is not widely used in the West, the clinical relevance of this meta-analysis is confined.

## Conclusion

This meta-analysis demonstrates that using UTI is associated with a shorter ICU LOS and MVD in CPB patients despite methodological limitations, and RFI generally showed a more favourable outcome with UTI treatment. According to the meta-regression and subgroup analysis, a higher UTI dosage seems to be correlated with better clinical outcomes. In summary, to better verify the beneficial effects of UTI treatment for CPB patients, further large, multicenter clinical trials and a standard dosing guideline are needed.

## Data Availability

Please contact the author for data requests.

## Abbreviations

UTI: Urinary trypsin inhibitor
CPB: Cardiopulmonary bypass
ICU: Intensive care unit
LOS: Length of stay
MVD: Mechanical ventilation duration
RFI: Renal failure incidence
CABG: Coronary artery bypass graft
AAR: Aortic arch replacement
VR: Valve replacement, including aortic valve replacement, mitral valve replacement
TAR: Total aortic arch replacement
MVR: Mitral valve replacement
DVR: Double (aortic and mitral) valve replacement
AVR: Aortic Valve Replacement
CI: Confidence interval
IV: Inverse variance
Std: Standardized mean difference
